# Placenta Accreta Spectrum Among Multiple Gestation: A Retrospective Analysis Based on a Chinese Population

**DOI:** 10.3389/fendo.2022.862785

**Published:** 2022-05-18

**Authors:** Zhirong Guo, Xueyan Han, Weiran Zheng, Huixia Yang, Jingmei Ma

**Affiliations:** ^1^Department of Obstetrics and Gynecology, Peking University First Hospital, Beijing, China; ^2^Beijing Key Laboratory of Maternal Fetal Medicine of Gestational Diabetes Mellitus, Peking University First Hospital, Beijing, China; ^3^Department of Medical Statistics, Peking University First Hospital, Beijing, China

**Keywords:** placenta accreta spectrum, multiple gestation, clinical characteristics, perioperative outcomes, assisted reproductive technology

## Abstract

**Background:**

It remained controversial whether women with multiple gestation are at higher risk of placenta accreta spectrum (PAS) disorders and large-scale studies are needed. This study aimed to assess whether PAS incidence is higher among women with multiple gestation than among singleton, as well as to compare the characteristics and outcomes of PAS in multiple and singleton gestation.

**Methods:**

Women who underwent cesarean section with live births at Peking University First Hospital from January 2015 to December 2020 were included. Demographic and clinical information was collected through chart review. Logistic regression models were used to analyze the associations between multiple gestation and PAS. The clinical characteristics and perioperative outcomes of PAS in multiple and singleton gestation were further compared.

**Results:**

Among the 14583 women included, 2.4% (352/14583) were diagnosed with PAS. PAS was slightly more prevalent among multiple gestations than among singletons (2.5% vs 2.4%, *P*=0.857). After adjusting for known risk factors and pregnancy complications, multiple gestation was associated with a higher risk of PAS (a*OR*=1.63, 95% *CI* 1.01-2.62). Among PAS patients, women who had multiple births had a significantly lower rate of previous cesarean deliveries (27.6% vs. 56.3%, *P*=0.003), placenta previa (17.2% vs. 56.3%, *P*<0.001) and invasive PAS (24.1% vs. 53.9, *P*=0.002) than singletons. There were no significant differences in perioperative outcomes between these two groups.

**Conclusion:**

Multiple gestation could be independently associated with an elevated risk of PAS. The clinical characteristics of PAS in the multiple and singleton gestation groups differed significantly in cesarean delivery history and placenta previa. The results of this study may inform guidelines on the screening, early detection and timely intervention of PAS patients among women with multiple births.

## Introduction

Placenta accreta spectrum (PAS) disorders are severe maternal complications characterized by abnormal adherence of the placental trophoblast to the uterine myometrium. It can be classified as placenta accreta (PA), placenta increta (PI) and placenta percreta (PP) according to the invasion depth of the placenta, and the latter two types are regarded as invasive PAS (1). The prevalence of PAS ranges from 0.01% to 1.1% (2, 3). A study using a nationally representative sample in the United States showed that the prevalence of PAS was 0.29% among women who underwent cesarean delivery with live births, and the rate increased by 2.1% per quarter year from 2015 to 2017 (4). Patients with PAS present considerably higher risks of catastrophic hemorrhage, hysterectomy, organ damage, consumptive coagulopathy and maternal death (2, 4–12). The known risk factors for PAS include placenta previa, prior cesarean delivery, prior uterine surgery and assisted reproductive technology (ART) (4, 13, 14).

It remains unclear whether women with multiple gestation are at higher risk for PAS. Miller et al. (15) found that women with twins conferred a 2.5 times higher risk for PAS than those with singleton gestation. However, Matsuzaki et al. (4) suggested that PAS was less prevalent among multiple births than singletons (*OR*=0.73). In view of the above controversy, Guo et al. (16) pointed out that the differences may be attributed to the study population, data source and model design. In addition, it is worth noting that both studies only used the International Classification of Diseases (ICD) codes to extract the diagnosis and subtype information of PAS; however, Jotwani et al. (17) observed that the overall accuracy of the ICD-10 for PAS was rather low, which stressed the importance of medical records in confirming PAS diagnosis. Inpatient records can also provide vital information on ante-partum diagnosis and care scheme, as well as the clinical confirmation of PAS diagnoses (by intrapartum observation or pathological results), which added precision to PAS diagnoses and provided the whole picture of peripartum PAS diagnosis and management.

In terms of peripartum outcomes, Shamshirsaz et al. (18) found that PAS patients with twins had a significantly higher red blood cell (RBC) transfusion volume than those with singletons, but they only included PAS patients who underwent cesarean hysterectomy. Therefore, a clearer understanding of the peripartum outcomes of PAS patients with multiple and singleton gestations is warranted.

This study aimed to explore the association between multiple gestation and PAS as well as to compare the peripartum outcomes of PAS patients with singletons and multiple births in a cohort of Chinese patients. The results of this study may shed light on the screening, early detection and timely intervention of PAS patients while informing clinicians and the research community on the characteristics of PAS patients with different gestation modes.

## Materials and Methods

### Data Collection

Live births that underwent cesarean section in the obstetrics department of the Peking University First Hospital from 2015 to 2020 were retrospectively included. Trained medical professionals reviewed electronic medical records and collected the clinical information. The PAS diagnoses were confirmed with the following diagnostic criteria through complete chart review.

### Diagnostic Criteria for PAS

All pregnancies managed by this center had gone through standardized complete placental evaluation. As suggested by the published guidelines (19, 20), pregnancies with a prior history of cesarean section and/or uterine surgery, low-lying placenta and placenta previa based on ultrasound findings would be screened more rigorously for PAS. The specific ultrasonic signs for PAS were adherent to previously reported guidelines (21, 22). MRI was used as an adjunct tool for the ultrasound in the following situations according to the obstetrician’s judgments: suspicions on PAS affecting adjacent pelvic organs, posterior placenta, maternal obesity affecting the diagnostic accuracy of ultrasound or inconclusive assessment on ultrasound. The diagnostic criteria for PAS were as follows: (a) pathological criteria (23): abnormal implantation of chorionic villi upon superficial or deep myometrium without a decidual layer based on microscopic diagnosis; (b) clinical diagnostic criteria (24) according to International Federation of Gynecology and Obstetrics (FIGO) guidelines: FIGO grade 1 (PA) presenting as placenta abnormally attached to the uterine muscle. The uterus shows no obvious distension over the placental bed, no placental tissue is seen invading through the surface of the uterus, and there is no or minimal neovascularity. In FIGO grade 2 (PI), significant amounts of hypervascularity in the uterine serosa without placenta invading through the uterine serosa were classified; placental tissue invading through the surface of the uterus and/or to parametrial regions or adjacent organs was classified as FIGO grade 3 (PP). We included patients with PAS if any of the criteria were met.

### Demographic and Clinical Information

The variables extracted for all the included patients included general data (delivery year, maternal age), information on maternal history (previous cesarean section), characteristics of the current pregnancy [gestational weeks, the use of assisted reproductive technology (ART)], pregnancy complications [placenta previa, multiple gestation, gestational diabetes mellitus (GDM), hypertensive disorders of pregnancy (HDP), intrahepatic cholestasis of pregnancy (ICP), premature rupture of membranes (PROM), idiopathic thrombocytopenic purpura (ITP), placental abruption, and abnormal amniotic fluid]. The full discharge medical records of PAS were further reviewed, and these variables were extracted additionally: detailed maternal history (previous abortion, endometrial injury), subtypes of PAS diagnosis (confirmed by the aforementioned diagnostic criteria), antenatal diagnosis (subtype and modality) and perioperative events (blood transfusion, hysterectomy, bladder injury, blood loss during operation, blood loss count, length of stay) were collected. The institutional ethics committee of Peking University First Hospital reviewed and approved this study [2019 (175)].

### Statistical Analysis

Continuous variables were presented as the mean ± standard deviation (SD). Independent sample *t* tests were used for group comparisons when the data conformed to a normal distribution. Nonnormally distributed data were presented as medians and interquartile ranges (IQRs), and rank-sum tests were used for descriptive analysis. Categorical data were presented as counts and percentages using chi-square tests or Fisher exact tests for group comparison.

Univariable and multivariable logistic regression models were used to investigate the association between PAS and multiple gestation. In multivariate logistic regression model 1, patient sociodemographic variables and known risk factors for PAS were adjusted, including year, maternal age ≥35, history of cesarean section, placenta previa and ART. In multivariate logistic regression model 2, pregnancy complications, including GDM, HDP, ICP, PROM, ITP, abnormal amniotic fluid, and placental abruption, were added. The *OR* value and 95% *CI* were reported. A two- sided *P* value <0.05 was considered significant for all analyses. The statistical analyses were conducted using Stata software (version 15.0; StataCorp, Texas, USA).

## Results

### Demographic and Clinical Characteristics Among Singletons and Multiple Gestations

The study included 14583 live births that underwent cesarean delivery, and 2.4% (352/14583) were diagnosed with PAS. The prevalence of PAS was 2.5% (29/1164) among multiple births and 2.4% (323/13419) among singleton pregnancies. Further analysis by PAS subtypes showed that the multiple births group had a significantly higher prevalence of PA (1.9% vs. 1.1%, *P*=0.018) and a significantly lower prevalence of invasive PAS (0.6% vs. 1.3%, *P*=0.040) than the singleton group. Women with multiple pregnancies were more likely to have ART (53.8% vs. 7.7%, *P*<0.001) but less likely to have a cesarean section history (11.2% vs. 33.6%, *P*<0.001). In terms of complications in current pregnancy, compared with singleton pregnancies, the multiple gestation group was more likely to suffer from HDP (23.4% vs. 12.2%, *P*<0.001), ICP (3.2% vs. 0.7%, *P*<0.001) and ITP (2.6% vs. 1.5%, *P*=0.004) and less likely to experience placenta previa (2.4% vs. 3.9%, *P*=0.012) and abnormal amniotic fluid (2.3% vs. 5.1%, *P*<0.001) (**Table 1**).

**Table 1:** Patients’ demographic and clinical characteristics (N=14583).

Characteristic [N (%)]	Multiple pregnancy (n=1164)		Singleton pregnancy (n=13419)	Statistic (χ^2^)	*P* value	
**General data**						
Delivery year						
After 2018 (2018-2020)	623 (53.5)		7074 (52.7)	0.279	0.597	
Advanced maternal age(≥35y)	414 (35.6)		5141 (38.3)	3.421	0.064	
**Previous history**						
Cesarean section*	130 (11.2)		4514 (33.6)	249.178	**<0.001**	
**Current pregnancy**						
ART *	626 (53.8)		1034 (7.7)	>1000	**<0.001**	
**Pregnancy complication**						
PAS	29 (2.5)		323 (2.4)	0.032	0.857	
Placenta accreta*	22 (1.9)		149 (1.1)	5.619	**0.018**	
Invasive PAS*	7 (0.6)		174 (1.3)	4.224	**0.040**	
Placenta previa*	28 (2.4)		519 (3.9)	6.343	**0.012**	
GDM	329 (28.3)		3794 (28.3)	0.001	0.995	
HDP*	272 (23.4)		1642 (12.2)	116.394	**<0.001**	
ICP*	37 (3.2)		100 (0.7)	68.157	**<0.001**	
Abnormal amniotic fluid*	27 (2.3)		686 (5.1)	17.962	**<0.001**	
PROM	167 (14.3)		2193 (16.3)	3.144	0.076	
Placental abruption	8 (0.7)		175 (1.3)	3.289	0.070	
ITP*	30 (2.6)		199 (1.5)	8.299	**0.004**	

PAS, placenta accreta spectrum; ART, assisted reproductive technology; GDM, gestational diabetes mellitus; HDP, hypertensive disorders of pregnancy; ICP, intrahepatic cholestasis of pregnancy; PROM, premature rupture of membranes, ITP, idiopathic thrombocytopenic purpura.

*Significant P values of <0.05. Bold font signified statistically significant P values.

### Associations Between Multiple Pregnancy and PAS

Univariate logistic regression showed that multiple gestation was associated with an increased likelihood of PAS (*OR*=1.04, 95% *CI* 0.71-1.52, *P*=0.857). After adjusting for delivery year, maternal age ≥35, history of cesarean section, placenta previa and ART in the multivariate logistic model (**Table 2**, Model 2), a significant association was found between multiple gestation and PAS (a*OR*=1.69, 95% *CI* 1.06-2.72, *P*=0.028). After adjusting for pregnancy complications in addition to Model 2, the significant association was preserved (a*OR*=1.63, 95% *CI* 1.01-2.62, *P*=0.044) (**Table 2**).

**Table 2:** Associations between multiple gestation and PAS (N=14583).

Unadjusted OR (95% CI)	Model 1	*P* value	Model 2	*P* value	Model 3	*P* value
			Adjusted OR (95% CI)		Adjusted OR (95% CI)	
Multiple gestation (ref. singleton pregnancy)	1.04 (0.71-1.52)	0.857	1.69 (1.06-2.72)	**0.028**	1.63 (1.01-2.62)	**0.044**

Model 1: Unadjusted, univariate analysis; Model 2: Adjusted for delivery year, maternal age≥35, the history of cesarean section, placenta previa and ART; Model 3: Adjusted for GDM, HDP, ICP, PROM, ITP, abnormal amniotic fluid, placental abruption plus variables adjusted in model 2. Bold font signified statistically significant P values.

### Clinical Characteristics and Perioperative Outcomes Among PAS of Multiple and Singleton Gestation

Among PAS patients, compared to singletons, multiple pregnancies were less likely to be multiparas (31.0% vs. 59.8%, *P*=0.001), have a history of previous cesarean deliveries (27.6% vs. 56.3%, *P*=0.003), be complicated with placenta previa (17.2% vs. 56.3%, *P*<0.001) in the current pregnancy and have a higher rate of undergoing ART (55.2% vs. 9.3%, *P*<0.001).

We examined the imaging modality and imaging reports before delivery and found that the overall antenatal diagnosis rate was 53.7% (189/352), and the rate was much higher in the singletons (56.0% vs 27.6%). For those with confirmed invasive PAS diagnosis (N=181), the overall antenatal diagnosis rate increased to 86.7% (157/181), and it was no longer significantly different between the singleton and multiple gestation PAS groups (86.8% vs. 85.7%) (**Table 3**). The overall antenatal MRI evaluation rate was 27.6% (97/352), and the rate was much lower in the multiple gestation group (10.3% vs. 29.1%). For the patients with the results of suspected PAS in the ultrasound test, 51.3% (97/189) had MRI results, and the lower trend in multiple gestation (37.5% vs. 51.9%) was preserved. For methods used to confirm PAS diagnosis, 17.9% (63/352) had confirmed pathological diagnosis (13.8% for multiple gestation, 18.3% for singleton), and others were diagnosed through intrapartum clinical diagnosis.

**Table 3:** Clinical characteristics and perioperative outcomes among PAS patients, stratified by singleton or multiple gestation (N=352).

Variables [N (%)]	Multiple pregnancy (n=29)	Singleton pregnancy (n=323)	Statistic	*P* value
**Characteristics**				
Delivery year				
After 2018 (2018-2020)	22 (75.9)	194 (60.1)	χ2 = 2.802	0.094
Advanced maternal age (≥35y)	16 (55.2)	158 (48.9)	χ2 = 0.417	0.519
**Maternal history**				
Gravidity≥3	7 (24.1)	132 (40.9)	χ2 = 3.117	0.077
Parity≥1*	9 (31.0)	193 (59.8)	χ2 = 11.240	**0.001**
Cesarean section*	8 (27.6)	182 (56.3)	χ2 = 8.974	**0.003**
Abortion	23 (79.3)	219 (67.8)	χ2 = 1.640	0.200
Endometrial injury	8 (27.6)	62 (19.2)	χ2 = 1.176	0.278
**Current pregnancy**				
Gestational weeks at delivery [weeks, M (P25~P75)]	36.0 (34.0-37.0)	36.0 (34.0-38.0)	Z=1.361	0.173
Placenta previa*	5 (17.2)	182 (56.3)	χ2 = 16.341	**<0.001**
ART*	16 (55.2)	30 (9.3)	–	**<0.001**
PAS subtypes*			χ2 = 9.417	**0.002**
Placenta accreta	22 (75.9)	149 (46.1)		
Invasive PAS	7 (24.1)	174 (53.9)		
Antenatal diagnosis (PAS)*	8 (27.6)	181 (56.0)	χ2 = 8.663	**0.003**
Antenatal diagnosis (Invasive PAS)^a^	6 (85.7)	151 (86.8)	−	1.000
PAS histopathological confirmation	4 (13.8)	59 (18.3)	χ2 = 0.362	0.547
Invasive PAS histopathological confirmation^a^	2 (28.6)	54 (31.0)	−	1.000
**Pregnancy complication**				
GDM	8 (27.6)	75 (23.2)	χ2 = 0.282	0.596
HDP*	9 (31.0)	26 (8.0)	–	**0.001**
ICP*	3 (10.3)	1 (0.3)	–	**0.002**
Abnormal amniotic fluid	1 (3.4)	10 (3.1)	–	1.000
PROM	3 (10.3)	12 (3.7)	–	0.117
Placenta abruption	0 (0.0)	2 (0.6)	–	1.000
ITP	1 (3.4)	1 (0.3)	–	0.158
**Peri-operational events**				
Blood transfusion	7 (24.1)	113 (35.0)	χ2 = 1.393	0.238
Hysterectomy (total or partial)	0 (0.0)	18 (5.6)	–	0.381
Bladder injury	0 (0.0)	5 (1.5)	–	0.500
Length of stay [days, M (P25~P75)]	6 (5-15)	7 (5-11)	Z=0.119	0.906
Blood loss volume [mL, M (P25~P75)]	800 (600-1200)	800 (500-1400)	Z=-0.177	0.859

PAS, placenta accreta spectrum; ART, assisted reproductive technology; GDM, gestational diabetes mellitus; HDP, hypertensive disorders of pregnancy; ICP, intrahepatic cholestasis of pregnancy; PROM, premature rupture of membranes, ITP, idiopathic thrombocytopenic purpura; US, ultrasound.

*Significant P values of <0.05. Bold font signified statistically significant P values.

^a^Calculated in the invasive PAS population. (N=181).

In terms of the comparison of PAS subtypes, the multiple gestation group had a significantly higher prevalence of placenta accreta (75.9% vs. 46.1%, *P*=0.002) and a lower prevalence of invasive PAS (24.1% vs. 53.9, *P*=0.002). PAS patients with multiple pregnancies were also more likely to have HDP (31.0% vs. 8.0%, *P*=0.001) and ICP (10.3% vs. 0.3%, *P*=0.002).

No differences were found in other pregnancy complications between groups (*P*>0.05). Comparison of perioperative outcomes showed that there were no differences in blood transfusion, hysterectomy, bladder injury, blood loss during operation, blood loss count or length of stay (*P*>0.05) (**Table 3**).

## Discussion

Our research revealed that multiple gestation was independently associated with the risk of PAS. Descriptive analyses showed that PAS among multiple births had a lower rate of previous cesarean section and placenta previa than singleton. There were no significant differences in perioperative outcomes of PAS between singleton and multiple pregnancies.

Our study found that multiple pregnancies may be associated with an increased risk of PAS, which was similar to the findings of Miller et al. (15). Jauniaux et al. (25) theorized that the rising incidence of placental implantation disorders (PIDs), including PAS, could be attributed to an increase in iatrogenic factors such as cesarean section and ART. These iatrogenic procedures may have an impact on the endometrium’s functional integrity, either directly or indirectly. The application of ART in multiple pregnancies may result in disturbance of interactions between the blastocyst and the endometrium, which might be the underlying mechanism for the association of ART and PAS. After adjusting for ART, multiple gestation remained an independent risk factor for PAS. Miller et al. (15) pointed out that in multiple pregnancies, an increased placental surface area is more likely to be implanted over the prior cesarean section scar, incurring PAS. It is evident that the specific mechanism behind the association between multiple gestation and PAS needs further study.

This study found that the overall antenatal diagnosis rate of PAS was 53.69% but only 27.6% among multiple gestations. This might be due to the relatively low prevalence of common PAS screening factors, such as placenta previa and prior cesarean deliveries, in the multiple gestation PAS population, which was also reported in other studies (15, 18). Additionally, more than 75% of PAS in the multiple gestation population was the placenta accreta subtype (compared to 36.1% in the singleton population), which could increase the difficulty in prenatal detection since most ultrasound signs were significantly more likely to be observed in severe percreta cases than mild accreta cases (26). Antenatal detection of PAS as well as management by a multidisciplinary team can reduce severe maternal morbidity (SMM) (10, 27). Since missed antenatal detection can result in catastrophic maternal and fetal outcomes (5), clarifying whether multiple gestation pose an increased risk for PAS could have important clinical implications.

The overall prenatal MRI use was low in this study, especially in the PAS patients with twin gestation. It was controversial regarding whether MRI evaluation could facilitate more prenatal detection of PAS. A recent systematic review comparing the additional clinical value of MRI found that both ultrasound and MRI showed comparable accuracy in the antenatal diagnosis of PAS and showed that the routine employment of MRI with high expense should not be recommended (28). Recent guideline (20) by Chinese experts also suggested that MRI should be used as an adjunct tool for patients who have already been screened on US and suspected to have PAS, rather than as a routine screening tool. However, whether additional MRI evaluation could increase the prenatal diagnostic accuracy of PAS in multiple gestation patients warrants further investigation.

The perioperative events of PAS were not significantly different between multiple and singleton gestation in this study. However, a multicenter study by Shamshirsaz et al. (18) showed that the PAS patients among multiple gestation received more units of RBC transfusion during and within 24 hours of surgery. Miller et al. (15) also found that multiple births with PAS were associated with a significantly higher rate of SMM (blood transfusion, hysterectomy, amniotic fluid embolism, disseminated intravascular coagulation, etc.) than singletons. In our study, the multiple gestation group had a significantly lower prevalence of invasive PAS, which could decrease SMM but it also had lower prenatal diagnosis rate which could cause more SMM. Therefore, whether twin gestation posed an additional risk of SMM in the PAS patients may need further investigation.

Our study’s strengths included the utilization of a multiyear database from a tertiary teaching hospital. The PAS patients in this study were pathologically or clinically confirmed instead of solely based on ICD codes, thus ensuring diagnostic accuracy for the included patients. Since the included patients were all managed in a single center by the same team, using a consistent surgical strategy with standardized pre, intra- and postoperative management protocols, the outcomes of this population were less likely to be confounded by the variations among caregivers.

There were also several limitations in the study. First, there could be some unmeasured biases that were inherent in retrospective studies. Future registered cohorts with prospective designs and data collection plans may provide higher-level evidence on the association. Second, the technology and healthcare quality evolved over a six-year observation time, although we did adjust for the year of delivery in the multivariate models. Third, the hysterectomy rate among PAS patients can be relatively low in our center, which could affect the generalizability of the results on patient outcomes. Finally, this was a single-center study despite its relatively large sample size. Multicenter studies could be designed to validate the current association and explore whether the results of the current study can be generalized.

## Conclusions

In conclusion, multiple gestation was associated with a higher risk of PAS. In the multiple births PAS group, traditional risk factors (placenta previa and previous cesarean deliveries) were identified less frequently, which posed challenges to prompt diagnosis of the disease. With the release of the three-child policy in China, the increase in pregnancies with previous cesarean section may lead to an increased incidence of PAS, which stresses the need to further understand this severe maternal complication. In addition, the development and increased utilization of ART technology can result in an increase in multiple gestations, which was the main focus of the current study. Further studies are warranted to explore the association between PAS and multiple births, whether multiple gestation is related to peripartum outcomes, and the mechanisms behind this phenomenon.

## Data Availability Statement

The datasets used in this study are available from the corresponding author upon reasonable request.

## Ethics Statement

The studies involving human participants were reviewed and approved by Ethics Committee of Peking University First Hospital. The ethics committee waived the requirement of written informed consent for participation.

## Author Contributions

ZG and XH collected and analyzed the data, prepared tables and figures, and drafted the paper; JM designed the research and revised the paper; WZ and HY provided clinical supervision. All authors have read and approved the final manuscript.

## Funding

This study was supported by the Strategic Collaborative Research Program of the Ferring Institute of Reproductive Medicine (Grant No. FIRMA 181104) and the National Key Technologies R&D program of China (Grant No. 2016YFC1000303).

## Conflict of Interest

The authors declare that the research was conducted in the absence of any commercial or financial relationships that could be construed as a potential conflict of interest.

## Publisher’s Note

All claims expressed in this article are solely those of the authors and do not necessarily represent those of their affiliated organizations, or those of the publisher, the editors and the reviewers. Any product that may be evaluated in this article, or claim that may be made by its manufacturer, is not guaranteed or endorsed by the publisher.
